# 2,2,2-Tris(pyrazol-1-yl)ethanol

**DOI:** 10.1107/S1600536811012931

**Published:** 2011-04-16

**Authors:** Craig C. McLauchlan, Brigette L. Smith, Reyhana S. Pippins, Brandon M. Nelson

**Affiliations:** a Department of Chemistry, Illinois State University, Campus Box 4160, Normal, IL 61790-4160, USA

## Abstract

The title compound TPE, C_11_H_12_N_6_O, was prepared by slow evaporation from diethyl ether. In the crystal, there is a hydrogen bond between the alcohol H atom and an N in the pyrazole ring of a neighboring mol­ecule.

## Related literature

For the original preparation, see: Reger *et al.* (2000 ▶). The title compound was prepared as part of our efforts to study tridentate scorpionate and psuedo-scorpionate ligands for coordination to vanadium, see: McLauchlan *et al.* (2004 ▶, 2009 ▶); McLauchlan & McDonald (2005 ▶, 2006 ▶). For coordination complexes with TPE, see: Sánchez-Méndez *et al.* (2004 ▶), Garcia-Orozco *et al.* (2006 ▶); Silva *et al.* (2009 ▶). For applications following substitution of the alcohol, see: Reger, Wright *et al.* (2001 ▶); Reger, Semeniuc *et al.* (2001 ▶); Reger & Grattan (2003 ▶); Pettinari & Pettinari (2005 ▶); Silva *et al.* (2009 ▶).
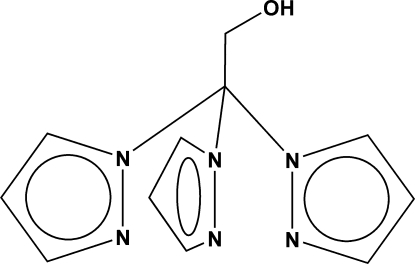


## Experimental

### 

#### Crystal data

C_11_H_12_N_6_O
*M* *_r_* = 244.27
Monoclinic, 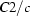

*a* = 19.6589 (14) Å
*b* = 11.5155 (8) Å
*c* = 12.4185 (18) Å
β = 125.740 (1)°
*V* = 2281.9 (4) Å^3^
*Z* = 8
Mo *K*α radiation
μ = 0.10 mm^−1^
*T* = 93 K
0.40 × 0.39 × 0.33 mm

#### Data collection

Bruker SMART APEX CCD diffractometer
Absorption correction: multi-scan (*SADABS*; Bruker, 2008 ▶) *T*_min_ = 0.699, *T*_max_ = 0.746
9505 measured reflections
2334 independent reflections
2187 reflections with *I* > 2σ(*I*)
*R*_int_ = 0.018

#### Refinement

*R*[*F* ^2^ > 2σ(*F* ^2^)] = 0.034
*wR*(*F* ^2^) = 0.087
*S* = 1.05
2334 reflections
167 parameters
H atoms treated by a mixture of independent and constrained refinement
Δρ_max_ = 0.27 e Å^−3^
Δρ_min_ = −0.25 e Å^−3^

### 

Data collection: *APEX2* (Bruker, 2008 ▶); cell refinement: *SAINT* (Bruker, 2008 ▶); data reduction: *SAINT*; program(s) used to solve structure: *SHELXTL* (Sheldrick, 2008 ▶); program(s) used to refine structure: *SHELXTL*; molecular graphics: *ORTEPIII* (Burnett & Johnson, 1996 ▶) and *Mercury* (Macrae *et al.*, 2008 ▶); software used to prepare material for publication: *SHELXTL* (Sheldrick, 2008 ▶).

## Supplementary Material

Crystal structure: contains datablocks global, I. DOI: 10.1107/S1600536811012931/dn2673sup1.cif

Structure factors: contains datablocks I. DOI: 10.1107/S1600536811012931/dn2673Isup2.hkl

Additional supplementary materials:  crystallographic information; 3D view; checkCIF report

## Figures and Tables

**Table 1:** Hydrogen-bond geometry (Å, °)

*D*—H⋯*A*	*D*—H	H⋯*A*	*D*⋯*A*	*D*—H⋯*A*
O1—H1*O*⋯N5^i^	0.851 (18)	2.003 (18)	2.8494 (13)	172.9 (16)

Symmetry code: (i) 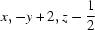.

**Fig. 1.**
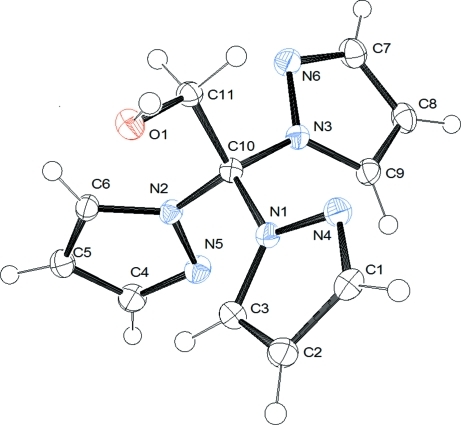
View of the molecular structure of the title compound showing 50% displacement ellipsoids (arbitrary spheres for the H atoms).

**Fig. 2.**
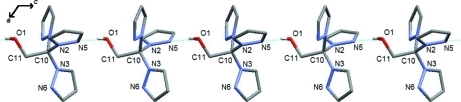
Several symmetry equivalent molecules of the title compound viewed along the b axis. Hydrogen bonds between the alcoholic O and the neighboring pyrazole N are indicated in light blue and are parallel to the c axis. The neighboring molecule is related by the symmetry code x, -y + 2, z - 1/2. Non-H-bonding hydrogen atoms have been removed for clarity.

## supplementary crystallographic information

### Comment

In our efforts to study tridentate scorpionate and psuedo-scorpionate ligands for coordination to vanadium (McLauchlan *et al.*, 2004; McLauchlan & McDonald, 2005; McLauchlan & McDonald, 2006; McLauchlan *et al.*, 2009), we have prepared and crystallized the title compound, tris-2,2,2-(1-pyrazolyl)ethanol, TPE, Figure 1. TPE had been reported by Reger *et al.* (2000) and used as a valuable tool for coordination chemistry owing to the ability to functionalize the alcohol (inter alia Reger, Wright *et al.*, 2001; Reger, Semeniuc *et al.*, 2001; Reger & Grattan, 2003; Pettinari & Pettinari, 2005; Silva *et al.*, 2009). Others have reported coordination complexes with TPE, including Sánchez-Méndez *et al.*(2004), Garcia-Orozco *et al.* (2006), and Silva *et al.* (2009). The structure of TPE shows evidence of hydrogen bonding between the alcoholic H and an N in the pyrazole ring of a neighboring molecule (Table 1) to form chains of TPE parallel to the *c* axis (Figure 2). The structural parameters are similar to those of that seen in the structure of TPE bound to Cu(II) by Silva *et al.* (2009).

### Experimental

The title compound was prepared using the method of Reger *et al.* (2000). Use of freshly sublimed KO*^t^*Bu was critical for reasonable yield (Silva *et al.*, 2009). Crystals suitable for diffraction study were obtained from slow evaporation of a diethyl ether solution of the product. Characterization data are in line with published data (Reger *et al.*, 2000).

### Refinement

The H atoms were geometrically placed (C—H = 0.93–0.97 Å) and refined as riding with *U*_iso_(H) = 1.2*U*_iso_(C) or 1.5*U*_eq_(methylene C). H atoms for the pyrazoles and the methylene unit were modeled in fixed positions whereas the alcoholic H (on O1) was allowed to refine freely.

### Crystal data

**Table:**

C_11_H_12_N_6_O	*F*(000) = 1024
*M**_r_* = 244.27	*D*_x_ = 1.422 Mg m^−^^3^
Monoclinic, *C*2/*c*	Melting point: 387(2) K
Hall symbol: -C 2yc	Mo *K*α radiation, λ = 0.71073 Å
*a* = 19.6589 (14) Å	Cell parameters from 6831 reflections
*b* = 11.5155 (8) Å	θ = 2.2–31.4°
*c* = 12.4185 (18) Å	µ = 0.10 mm^−^^1^
β = 125.740 (1)°	*T* = 93 K
*V* = 2281.9 (4) Å^3^	Block, colorless
*Z* = 8	0.40 × 0.39 × 0.33 mm

### Data collection

**Table:**

Bruker SMART APEX CCD diffractometer	2334 independent reflections
Radiation source: fine-focus sealed tube	2187 reflections with *I* > 2σ(*I*)
graphite	*R*_int_ = 0.018
ω scans	θ_max_ = 26.4°, θ_min_ = 2.2°
Absorption correction: multi-scan (*SADABS*; Bruker, 2008)	*h* = −24→23
*T*_min_ = 0.699, *T*_max_ = 0.746	*k* = −14→14
9505 measured reflections	*l* = −15→15

### Refinement

**Table:**

Refinement on *F*^2^	Primary atom site location: structure-invariant direct methods
Least-squares matrix: full	Secondary atom site location: difference Fourier map
*R*[*F*^2^ > 2σ(*F*^2^)] = 0.034	Hydrogen site location: inferred from neighbouring sites
*wR*(*F*^2^) = 0.087	H atoms treated by a mixture of independent and constrained refinement
*S* = 1.05	*w* = 1/[σ^2^(*F*_o_^2^) + (0.0454*P*)^2^ + 1.8611*P*] where *P* = (*F*_o_^2^ + 2*F*_c_^2^)/3
2334 reflections	(Δ/σ)_max_ = 0.001
167 parameters	Δρ_max_ = 0.27 e Å^−^^3^
0 restraints	Δρ_min_ = −0.25 e Å^−^^3^

### Special details

**Table:**

Geometry. All e.s.d.'s (except the e.s.d. in the dihedral angle between two l.s. planes) are estimated using the full covariance matrix. The cell e.s.d.'s are taken into account individually in the estimation of e.s.d.'s in distances, angles and torsion angles; correlations between e.s.d.'s in cell parameters are only used when they are defined by crystal symmetry. An approximate (isotropic) treatment of cell e.s.d.'s is used for estimating e.s.d.'s involving l.s. planes.
Refinement. Refinement of *F*^2^ against ALL reflections. The weighted *R*-factor *wR* and goodness of fit *S* are based on *F*^2^, conventional *R*-factors *R* are based on *F*, with *F* set to zero for negative *F*^2^. The threshold expression of *F*^2^ > σ(*F*^2^) is used only for calculating *R*-factors(gt) *etc*. and is not relevant to the choice of reflections for refinement. *R*-factors based on *F*^2^ are statistically about twice as large as those based on *F*, and *R*- factors based on ALL data will be even larger. H atoms for the pyrazoles and the methylene unit were modeled in fixed positions whereas the alcoholic H (on O1) was allowed to refine freely.

### Fractional atomic coordinates and isotropic or equivalent isotropic displacement parameters (Å^2^)

**Table:**

	*x*	*y*	*z*	*U*_iso_*/*U*_eq_	
O1	0.16215 (5)	0.93915 (7)	0.05201 (8)	0.01904 (19)	
N1	0.14888 (6)	1.06979 (8)	0.22818 (9)	0.0163 (2)	
N2	0.18638 (6)	0.87863 (8)	0.30788 (9)	0.0161 (2)	
N3	0.29052 (6)	1.02830 (8)	0.40204 (9)	0.0169 (2)	
N4	0.16341 (6)	1.17862 (8)	0.20317 (10)	0.0198 (2)	
N5	0.17186 (6)	0.88180 (8)	0.40312 (9)	0.0207 (2)	
N6	0.36644 (6)	0.98761 (9)	0.43963 (10)	0.0225 (2)	
C1	0.08823 (7)	1.22859 (10)	0.13521 (11)	0.0196 (2)	
H1A	0.0782	1.3066	0.1046	0.024*	
C2	0.02568 (7)	1.15312 (10)	0.11394 (11)	0.0214 (2)	
H2A	−0.0324	1.1691	0.0684	0.026*	
C3	0.06644 (7)	1.05131 (10)	0.17310 (11)	0.0198 (2)	
H3A	0.0418	0.9812	0.1753	0.024*	
C4	0.13735 (8)	0.77969 (10)	0.39302 (12)	0.0231 (3)	
H4A	0.1210	0.7557	0.4480	0.028*	
C5	0.12786 (7)	0.71147 (10)	0.29158 (12)	0.0221 (2)	
H5A	0.1045	0.6358	0.2652	0.027*	
C6	0.15956 (7)	0.77737 (9)	0.23876 (11)	0.0180 (2)	
H6A	0.1623	0.7563	0.1674	0.022*	
C7	0.42096 (8)	1.04416 (11)	0.55168 (12)	0.0242 (3)	
H7A	0.4800	1.0341	0.6022	0.029*	
C8	0.38124 (8)	1.12033 (10)	0.58653 (11)	0.0231 (3)	
H8A	0.4068	1.1699	0.6617	0.028*	
C9	0.29742 (7)	1.10762 (10)	0.48837 (11)	0.0199 (2)	
H9A	0.2527	1.1469	0.4821	0.024*	
C10	0.21515 (7)	0.98322 (9)	0.28018 (10)	0.0156 (2)	
C11	0.23604 (7)	0.95939 (10)	0.17984 (10)	0.0168 (2)	
H11A	0.2662	1.0269	0.1771	0.020*	
H11B	0.2732	0.8908	0.2089	0.020*	
H1O	0.1605 (10)	0.9921 (15)	0.0027 (17)	0.033 (4)*	

### Atomic displacement parameters (Å^2^)

**Table:**

	*U*^11^	*U*^22^	*U*^33^	*U*^12^	*U*^13^	*U*^23^
O1	0.0198 (4)	0.0210 (4)	0.0151 (4)	−0.0026 (3)	0.0094 (3)	0.0001 (3)
N1	0.0177 (5)	0.0141 (4)	0.0187 (5)	−0.0005 (3)	0.0116 (4)	−0.0007 (3)
N2	0.0192 (5)	0.0152 (4)	0.0161 (4)	0.0000 (3)	0.0115 (4)	−0.0003 (3)
N3	0.0167 (5)	0.0166 (4)	0.0171 (5)	−0.0006 (3)	0.0096 (4)	−0.0015 (4)
N4	0.0228 (5)	0.0154 (5)	0.0249 (5)	0.0002 (4)	0.0160 (4)	0.0017 (4)
N5	0.0278 (5)	0.0207 (5)	0.0192 (5)	−0.0005 (4)	0.0169 (4)	0.0002 (4)
N6	0.0168 (5)	0.0238 (5)	0.0220 (5)	0.0014 (4)	0.0087 (4)	−0.0031 (4)
C1	0.0235 (6)	0.0177 (5)	0.0190 (5)	0.0032 (4)	0.0132 (5)	0.0008 (4)
C2	0.0176 (5)	0.0227 (6)	0.0208 (6)	0.0010 (4)	0.0095 (5)	−0.0018 (4)
C3	0.0175 (5)	0.0199 (5)	0.0215 (5)	−0.0029 (4)	0.0111 (5)	−0.0029 (4)
C4	0.0292 (6)	0.0211 (6)	0.0255 (6)	−0.0015 (5)	0.0196 (5)	0.0024 (4)
C5	0.0246 (6)	0.0168 (5)	0.0266 (6)	−0.0023 (4)	0.0159 (5)	−0.0010 (4)
C6	0.0189 (5)	0.0157 (5)	0.0187 (5)	0.0000 (4)	0.0106 (5)	−0.0019 (4)
C7	0.0197 (6)	0.0265 (6)	0.0203 (6)	−0.0025 (5)	0.0081 (5)	−0.0023 (5)
C8	0.0267 (6)	0.0234 (6)	0.0184 (6)	−0.0069 (5)	0.0128 (5)	−0.0045 (4)
C9	0.0254 (6)	0.0189 (5)	0.0205 (5)	−0.0032 (4)	0.0163 (5)	−0.0031 (4)
C10	0.0158 (5)	0.0147 (5)	0.0162 (5)	−0.0004 (4)	0.0093 (4)	−0.0010 (4)
C11	0.0166 (5)	0.0188 (5)	0.0161 (5)	−0.0012 (4)	0.0102 (4)	−0.0009 (4)

### Geometric parameters (Å, °)

**Table:**

O1—C11	1.4108 (13)	C2—C3	1.3669 (17)
O1—H1O	0.851 (18)	C2—H2A	0.9500
N1—C3	1.3582 (14)	C3—H3A	0.9500
N1—N4	1.3610 (13)	C4—C5	1.4015 (17)
N1—C10	1.4578 (13)	C4—H4A	0.9500
N2—C6	1.3586 (14)	C5—C6	1.3683 (16)
N2—N5	1.3698 (13)	C5—H5A	0.9500
N2—C10	1.4546 (14)	C6—H6A	0.9500
N3—C9	1.3535 (14)	C7—C8	1.4005 (18)
N3—N6	1.3607 (13)	C7—H7A	0.9500
N3—C10	1.4613 (14)	C8—C9	1.3696 (17)
N4—C1	1.3307 (15)	C8—H8A	0.9500
N5—C4	1.3263 (15)	C9—H9A	0.9500
N6—C7	1.3292 (16)	C10—C11	1.5475 (15)
C1—C2	1.3992 (16)	C11—H11A	0.9900
C1—H1A	0.9500	C11—H11B	0.9900
			
C11—O1—H1O	105.9 (11)	C6—C5—H5A	127.4
C3—N1—N4	112.15 (9)	C4—C5—H5A	127.4
C3—N1—C10	127.64 (9)	N2—C6—C5	106.90 (10)
N4—N1—C10	118.58 (9)	N2—C6—H6A	126.6
C6—N2—N5	111.62 (9)	C5—C6—H6A	126.6
C6—N2—C10	128.49 (9)	N6—C7—C8	112.10 (11)
N5—N2—C10	119.22 (9)	N6—C7—H7A	124.0
C9—N3—N6	112.23 (9)	C8—C7—H7A	124.0
C9—N3—C10	129.19 (9)	C9—C8—C7	104.84 (10)
N6—N3—C10	118.58 (9)	C9—C8—H8A	127.6
C1—N4—N1	103.86 (9)	C7—C8—H8A	127.6
C4—N5—N2	104.35 (9)	N3—C9—C8	106.80 (10)
C7—N6—N3	104.04 (9)	N3—C9—H9A	126.6
N4—C1—C2	112.28 (10)	C8—C9—H9A	126.6
N4—C1—H1A	123.9	N2—C10—N1	107.50 (8)
C2—C1—H1A	123.9	N2—C10—N3	110.38 (9)
C3—C2—C1	104.87 (10)	N1—C10—N3	108.60 (8)
C3—C2—H2A	127.6	N2—C10—C11	111.49 (8)
C1—C2—H2A	127.6	N1—C10—C11	110.70 (9)
N1—C3—C2	106.78 (10)	N3—C10—C11	108.15 (9)
N1—C3—H3A	126.6	O1—C11—C10	110.62 (9)
C2—C3—H3A	126.6	O1—C11—H11A	109.5
N5—C4—C5	111.97 (10)	C10—C11—H11A	109.5
N5—C4—H4A	124.0	O1—C11—H11B	109.5
C5—C4—H4A	124.0	C10—C11—H11B	109.5
C6—C5—C4	105.14 (10)	H11A—C11—H11B	108.1
			
C3—N1—N4—C1	2.18 (12)	C6—N2—C10—N1	105.80 (12)
C10—N1—N4—C1	168.76 (9)	N5—N2—C10—N1	−63.96 (12)
C6—N2—N5—C4	1.32 (12)	C6—N2—C10—N3	−135.91 (11)
C10—N2—N5—C4	172.70 (9)	N5—N2—C10—N3	54.33 (12)
C9—N3—N6—C7	−0.22 (13)	C6—N2—C10—C11	−15.69 (15)
C10—N3—N6—C7	−179.92 (10)	N5—N2—C10—C11	174.56 (9)
N1—N4—C1—C2	−1.23 (12)	C3—N1—C10—N2	−20.24 (14)
N4—C1—C2—C3	−0.10 (13)	N4—N1—C10—N2	175.52 (9)
N4—N1—C3—C2	−2.31 (13)	C3—N1—C10—N3	−139.67 (11)
C10—N1—C3—C2	−167.39 (10)	N4—N1—C10—N3	56.09 (12)
C1—C2—C3—N1	1.41 (12)	C3—N1—C10—C11	101.74 (12)
N2—N5—C4—C5	−1.10 (13)	N4—N1—C10—C11	−62.50 (12)
N5—C4—C5—C6	0.51 (14)	C9—N3—C10—N2	−91.92 (13)
N5—N2—C6—C5	−1.04 (12)	N6—N3—C10—N2	87.72 (11)
C10—N2—C6—C5	−171.42 (10)	C9—N3—C10—N1	25.69 (15)
C4—C5—C6—N2	0.32 (13)	N6—N3—C10—N1	−154.67 (9)
N3—N6—C7—C8	0.03 (14)	C9—N3—C10—C11	145.87 (11)
N6—C7—C8—C9	0.15 (14)	N6—N3—C10—C11	−34.49 (13)
N6—N3—C9—C8	0.32 (13)	N2—C10—C11—O1	70.77 (11)
C10—N3—C9—C8	179.98 (10)	N1—C10—C11—O1	−48.84 (12)
C7—C8—C9—N3	−0.28 (13)	N3—C10—C11—O1	−167.70 (8)

### Hydrogen-bond geometry (Å, °)

**Table:**

*D*—H···*A*	*D*—H	H···*A*	*D*···*A*	*D*—H···*A*
O1—H1O···N5^i^	0.851 (18)	2.003 (18)	2.8494 (13)	172.9 (16)

Symmetry codes: (i) *x*, −*y*+2, *z*−1/2.
